# Effect of Lactobacilli on Paracellular Permeability in the Gut

**DOI:** 10.3390/nu3010104

**Published:** 2011-01-12

**Authors:** Siv Ahrne, Marie-Louise Johansson Hagslatt

**Affiliations:** Division of Applied Nutrition, Department of Food Technology, Engineering and Nutrition, Lund University, P.O. Box 124, SE-22100 Lund, Sweden; Email: marie-louise.hagslatt@insatnet.nu

**Keywords:** intestinal lactobacilli, probiotics, paracellular permeability, tight junctions

## Abstract

Paracellular permeability is determined by the complex structures of junctions that are located between the epithelial cells. Already in 1996, it was shown that the human probiotic strain *Lactobacillus plantarum* 299v and the rat-originating strain *Lactobacillus reuteri* R2LC could reduce this permeability in a methotrexate-induced colitis model in the rat. Subsequently, many animal models and cell culture systems have shown indications that lactobacilli are able to counteract increased paracellular permeability evoked by cytokines, chemicals, infections, or stress. There have been few human studies focusing on the effect of lactobacilli on intestinal paracellular permeability but recently it has been shown that they could influence the tight junctions. More precisely, short-term administration of *L. plantarum* WCSF1 to healthy volunteers increased the relocation of occludin and ZO-1 into the tight junction area between duodenal epithelial cells.

## 1. Introduction

**Figure 1:**
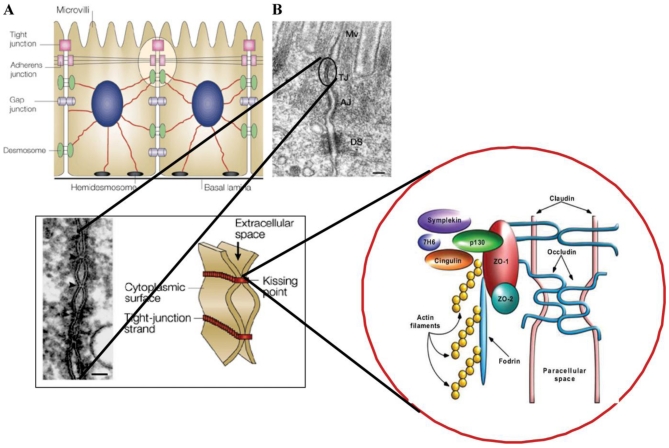
(**A**) Macroscopic arrangement and (**B**) microscopic composition of intercellular tight junctions; reproduced with permission from John Wiley and Sons [21].

A thin layer of epithelial cells, extending from the sphincter of the esophagus to the anus, transports nutrients and water from the intestinal content while also functioning as a barrier to antigenic molecules from food and commensal as well as pathogenic bacteria. The epithelium confers selective permeability through either transcellular or paracellular routes. Transfer of small molecules such as short-chain fatty acids, amino acids, electrolytes and sugars are transporter-mediated through cells while medium-sized (up to approximately 600 Da *in vivo* and 10 kDa in cell lines) hydrophilic compounds are transferred paracellularly. Protein-sized molecules are normally prohibited from transport via the paracellular route (Figure 1) [1,2]. 

The paracellular barrier function is brought about by complex junctional structures, the tight junctions (TJs) located apically between the enterocytes and the more basolateral adherens junctions, the desmosomes and the gap junctions. These complexes have long been known to transport fluids and molecules and to structurally anchor adjacent cells together. However, the junctional complexes are now known to also take part in transfer of external stimuli to the epithelial cells governing their proliferation and differentiation [3]. The current view is that the function of TJs is what is mainly responsible for the paracellular permeability [4].

Tight junctions are made up of transmembrane proteins such as occludins, junctional adhesion molecules (JAM) and claudins with an intra-cellular connection to the zonulins, which are members of the zonula occludens (ZO) family. These, in turn, are anchored to the cell’s actinomyosin cytoskeleton and the result is a structure that not only provides the epithelium with a barrier function but also, by rapid assembly and disassembly, changes its permeability upon different stimuli (Figure 1) [5,6]. Moreover, they maintain the polarity of the epithelial cell, making it impossible for lipids and proteins, directed towards the luminal side, to move basolaterally [2]. The function of the TJs is dependent on levels and localization of junction proteins and also regulated by phosphorylation of the TJ proteins and of the myosin light chain (MLC). The latter is brought about by different kinases and phosphorylases, and results in the fine-tuning of the paracellular permeability according to the cellular environment [5]. 

Altered intestinal permeability, as a result of increased paracellular permeability, enhanced transcellular transport or even loss of epithelial cells, plays an important role in the pathogenesis in several critical conditions such as burns, major trauma and sepsis [6,7,8]. Impaired gut barrier function may take days to restore and could eventually lead to an increased translocation of intestinal bacteria into the body [9]. Under these circumstances the gut has been called “the undrained abscess” [10], and has been regarded as a driving force towards multiple organ failure [11,12]. Bacterial translocation, which has been recorded in numerous animal models, is defined as passage of viable bacteria from the gastrointestinal tract to extra intestinal sites and could take place between, as well as through the epithelial cells. However, for obvious reasons, measurement of translocation in humans is difficult and so its significance in the clinical situation remains controversial [13].

Increased permeability of the intestinal epithelium, with alterations of the paracellular pathway and also in the transcytotic uptake of peptides, is also mentioned as part of the pathophysiology of many less dramatic diseases [2,14]. Mediators of inflammation such as reactive oxygen species (ROS), endotoxin (lipopolysaccharide; LPS) and cytokines can induce disruption of TJs and thereby increase the paracellular permeability [15]. Abnormal intestinal barrier function plays a pivotal role in inflammatory bowel disease (IBD) [16]. Type-1 diabetes and coeliac disease are examples of auto‑immune situations where increased paracellular permeability has been implicated in the development of disease [17]. In addition, a barrier dysfunction in the colonic mucosa of Irritable Bowel Syndrome (IBS) patients that was the result of increased paracellular permeability, presumably by an altered expression of ZO‑1, has been reported [18]. Moreover, stress is believed to contribute to induction of IBS as well as recurrence of intestinal inflammation and onset of food allergies, and stressful stimuli can increase paracellular permeability [19]. For gastrointestinal diseases, altered intestinal permeability could certainly be a result of disease progression but there is evidence that it might be a primary event. Increased paracellular permeability is, for instance, predictive of relapse in inactive IBD patients and present in first-degree asymptotic relatives of patients with celiac disease [1].

Members of the *Lactobacillus* genus are a minor part of the normal intestinal microbiota in the colon but a much greater component higher up in the gut. The species that are most commonly found in this niche are *Lactobacillus paracasei*, *Lactobacillus salivarius*, *Lactobacillus rhamnosus*, *Lactobacillus fermentum* and *Lactobacillus plantarum* [20]. Members of these species have also been encountered as having probiotic effects and have been challenged as such in many *in vitro* as well as *in vivo* studies. In this article, we are trying to picture the effects that this genus might have on the paracellular permeability in the gut.

## 2. Effects in Animal Models

### 2.1. Methotrexate-Induced Colitis

Methotrexate is a cytotoxic drug that interferes with folate metabolism and so decreases *de novo* synthesis of thymidine and purine, resulting in inhibition of rapidly dividing cells such as cancer cells. The fact that intestinal epithelial cells also divide rapidly renders them vulnerable to methotrexate. The damage is characterized by decreased intestinal barrier function, infiltration to the area of neutrophils and macrophages and thereby an increasing amount of ROS at the site of injury. In the methotrexate-induced colitis model, ROS generated by chemotherapy is the first step leading to increased intestinal permeability and intestinal inflammation [22]. ROS has been reported to activate tyrosine kinase and phosphatidylinositol-3-kinase and alter the composition of the tight junctions that will affect the paracellular permeability [23,24].

Administration of *L. plantarum* DSM 9843 (299v) and *Lactobacillus reuteri* R2LC in a methotrexate-induced colitis model in the rat decreased intestinal permeability, as measured in the proximal as well as distal ileum and in colon by Cr-EDTA clearance. This molecule is found in increased quantities in urine while the gut permeability is enhanced and regarded as a probe that could be used for such measurements for both the small intestine and the colon. Translocation to mesenteric lymph nodes (MLNs), liver, spleen and arterial blood and endotoxin levels in plasma were decreased in these treatment groups and intestinal microflora was affected, showing less *Enterobacteriaceae* and Gram-negative anaerobes, especially in the rats treated with *L. plantarum* [25]. Pre-treatment for one week before administration of methotrexate, using cow’s milk fermented with *Lactobacillus johnsonii* and sheep’s milk fermented with *Lactobacillus bulgaricus* and *Streptococcus thermophilus*, was shown to improve small intestinal barrier function as determined by the lactulose/mannitol ratio [26]. Together with rhamnose, lactulose and mannitol are sugars that are often used as probes to monitor paracellular permeability, although they can only be used to illustrate the small intestine as they are broken down by the microbiota in the colon.

### 2.2. Liver Injury Induced by D-Galactosamine

The outcome of liver injury induced by D-galactosamine resembles clinical features of fulminant liver failure in many respects [27]. D-galactosamine is a hepatocyte-specific inhibitor of RNA synthesis and has been shown to increase the sensitivity to LPS and to the pro-inflammatory cytokine tumor necrosis factor alpha (TNF-α) [28]. Increased gut permeability after insult has been seen in the distal ileum and cecum by measuring the passage of Cr-EDTA, together with an increased amount of translocating bacteria in mesenteric lymphnodes and liver as compared to control animals without any injury [29]. As endotoxin is implicated in the progression of D-galactosamine-induced liver injury [30], simultaneous administration of LPS from *Escherichia coli* is often used in order to increase the severity of the model. In such settings, intestinal bacteria were seen to invade the mucosa nine hours after the insult and a simultaneous collapse of TJ integrity was observed in the mice. The authors discussed the possibility that destroyed polarity may have facilitated the translocation between epithelial cells [31].

The probiotic mixture VSL#3 (one strain each of *L. plantarum*, *Lactobacillus casei*, *Lactobacillus delbrueckii* subsp. *bulgaricus*, *Lactobacillus acidophilus*, *Bifidobacterium breve*, *Bifidobacteriun infantis*, *Bifidobacterium longum* and *Streptococcus salivarius* subsp. *thermophilus*) was challenged for its effect in an acute liver injury model in the mice induced by D-galactosamine and LPS. The diseased control group of mice showed a breakdown of colonic barrier function, as measured by mannitol movement in the Ussing chamber, which is a device where intestinal pieces can be mounted and the flux of different molecules from the mucosal to the serosal side monitored. The increase in permeability of mannitol was concomitant with an increase of pro-inflammatory cytokines in colon tissue as well as bacterial translocation to the liver and hepatic injury. In a peroxisome proliferator-activated receptor-gamma (PPAR-γ) dependent manner, the pre-treatment by probiotics before insult prevented the permeability increase as well as the elevated translocation and the liver injury [32]. Although the probiotic used in that study was a mixture of strains from different species as well as genera, pre-treatment by a single strain of lactobacilli has given similar outcomes in this model. Decreased translocation of bacteria to mesenteric lymph nodes and liver, as well as to arterial and portal blood, increased DNA and RNA content of mucosal tissue, and reduced hepatocyte damage (as indicated by a decrease in the release of liver enzymes) was obtained after pre-treatment with *L. plantarum* 299v [33,34]. 

### 2.3. Dextran Sulfate-Induced Colitis

Increased epithelial permeability is intimately linked to the inflammation process in IBD. The etiology of IBD, mainly ulcerative colitis (UC) and Crohn’s disease (CD), is not known but it has been proposed that the excessive immunological reactivity partly depends on the increased intestinal permeability [17]. Even though it has been shown that there are alterations in the TJ complex, concomitant to increased permeability during inflammation, the question remains as to whether the change in permeability is a prerequisite of inflammation or *vice versa* [35].

Administration of dextran sulfate sodium (DSS) to rodents has been widely used as a model for inducing acute and chronic colitis, as both clinical and histopathological outcomes resemble those of human ulcerative colitis [36]. The DSS model for mice shows that one of the earliest events is the loss of ZO-1. This is followed by an increase in intestinal permeability, followed by higher scores in disease activity index (DAI) as well as inflammation [35]. 

In a recent study of DSS given to rats, the probiotic mixture of strain mixture VSL#3 was shown to counteract apoptosis and protect against increased intestinal permeability by up-regulation of the expression of proteins of the tight junctions, namely of occludin, ZO-1 and claudines 1–5 [37]. Again, the treatment comprised a mixture of strains of different species and genera as outlined above, but effects of single strains of lactobacilli in this model have also been shown. *L. plantarum* DSM 9843 (299v), *L. plantarum* 15313, and *L. fermentum* 35D have also been shown to counteract the increase in DAI after DSS treatment [38,39,40]. When colon mucosal infiltration of neutrophils and oxidative stress were measured as myeloperoxidase (MPO) and malondialdehyde (MDA), respectively, *L. plantarum* 15313 and *L. fermentum* 35D significantly decreased the MPO, while *L. plantarum* DSM 9843 and *L. plantarum* 15313 were able to reduce the MDA [39,40]. 

The effect of both heat-killed and live *L. rhamnosus* OLL2838 on DSS-treated BALB/c mice has recently been studied in detail. While it suppressed both weight-loss and promoted colon length it also restored intestinal barrier function. According to the authors, the protection against increased mucosal permeability observed “may at least partially be because of the increased expression of zonula occludens-1 (4.8 fold) and myosin light-chain kinase (3.1 fold) in intestinal epithelial cells isolated from mice of the heat-killed group” [41]. Myosin light-chain phosphorylation is regarded as leading to contraction of the cytoskeleton and increased paracellular permeability [4]. However, DSS treatment and inhibition of myosin light-chain kinase (MLCK) have been shown to induce apoptosis of intestinal epithelial cells. Accordingly, normalization by *L. rhamnosus* OLL2838 of the MLCK activity, as mentioned above, might have contributed to barrier restoration [41].

### 2.4. Alcohol-Induced Injury

Alcohol intoxication is associated with an increased translocation of bacteria from the gut as well as endotoxemia [42], which could partly depend on gut leakiness mediated by increased oxidative stress [43]. The transcription factor NF-κB has been demonstrated to be up-regulated by alcohol [44] and thereby increase the inducible nitric oxide synthase and thus the production of reactive nitrogen species. This oxidative stress promotes carbonylation and nitrotyrosination of the actin and microtubule cytoskeletons, leading to loss in tight junction integrity [45]. 

In a recent study of alcohol injury in the rat, *L. rhamnosus* GG reduced the alcohol-induced intestinal leakage and decreased oxidative stress in both small intestine and colon, as shown by lower carbonyl and nitrotyrosine levels in the probiotic treatment group. Moreover, MPO as a marker of neutrophil infiltration and inflammation in colon was significantly reduced by the administration of *L. rhamnosus* GG [43].

### 2.5. Stress-Induced Models

Stress is known to affect gastrointestinal functions, such as gut motility, and to impair the barrier function by increasing the paracellular permeability [20]. Animal models of both acute and chronic stress have been developed with manifestations of enhanced bacterial adherence to and internalization of the gut wall, increased translocation and altered gut permeability [46,47]. 

In a chronic stress model induced by water avoidance, elevated base line short-circuit current and increased conductance in the Ussing chamber were noted along with increased bacterial adhesion, penetration and translocation. The baseline short circuit current and transepithelial electrical resistance (TER) are parameters that could be monitored in the Ussing chamber and that depend on the movement of ions across the paracellular pathway. The probiotic strains *L. rhamnosus* R0011 (95%) and *Lactobacillus helveticus* R0052 (5%) had no effect on intestinal permeability as such but counteracted the elevated base-line short-circuit current and the translocation of bacteria to mesenteric lymph nodes [46]. The same *Lactobacillus* preparation was used in a rat model of maternal stress where the pups were separated from the dam for three hours per day from day 4 to day 19. The stressed animals without treatment showed increased stimulated short-circuit current and elevated permeability to horseradish peroxidase. The adhesion to and penetration of bacteria to epithelial cells was increased by the stress whereas the lactobacilli counts were less than in non-stressed pups. Probiotic treatment ameliorated these abnormalities. In the meantime, the elevated serum corticosterone levels obtained by maternal stress were restored at “non-stressed” levels in the probiotic treatment group. The authors conclude that “probiotics improve gut dysfunction induced by maternal separation, at least in part by normalization of HPA axis activity” [48]. 

Hypersensitivity to distension has been connected to an altered colonic paracellular permeability resulting from epithelial cell cytoskeleton contraction through myosin light chain kinase activation [49]. Visceral sensitivity and paracellular permeability were monitored using maternal stress as well as partial restraint stress (PRS; 2 h/day restriction of body movement) with the aim of testing the counteractive effect of three different probiotic strains on the adverse manifestations. *L. paracasei* NCC2461 but not *Bifidobacterium lactis* NCC362 or *Lactobacillus johnsonii* NCC533 improved stress-induced visceral pain and restored normal gut permeability in the maternal stress model. Furthermore, in order for this strain to prevent PRS-induced hypersensitivity, both bacteria and bacterial products were required [50]. A member of the species *Lactobacillus farciminis*, strain CIP 103136, could prevent the acute stress-induced hypersensitivity to distension and the increase in paracellular permeability provoked in a similar PRS model. Treatment by this strain counteracted the otherwise increased phosphorylation of the myosin light chain that is characteristic for this model. Nitric oxide production of the strain was implicated as playing a role [51]. 

### 2.6. Healthy Animals

There is no doubt that evidence for the effect of lactobacilli on paracellular permeability in animal models is increasing, but studies in healthy animals are few. However, it has been shown in Sprague-Dawley rats that administration of *L. plantarum* 299v counteracts the otherwise increased passage of mannitol across the small intestinal wall induced by a non-pathogenic strain of *E. coli*. This was measured in the Ussing chamber and, interestingly, the prevention was only obtained when the lactobacilli were administered in the drinking water but not via tube feeding twice a day [52]. The authors discuss the possibility of the received quantity being responsible for this discrepancy. Around four times more lactobacilli were ingested via the water as compared to tube feeding but, in light of the stress-evoked manifestations on permeability mentioned above, the fact that the animals were tube-fed twice a day could have been of importance for the outcome. In any case, *L. plantarum* 299v administered for one week in the drinking water could protect from *E. coli*-induced permeability in perfectly healthy animals.

## 3. Effects in Different Cell Systems

In order to find effects and to understand underlying mechanisms of lactobacilli on paracellular permeability, monolayers of different epithelial cell lines have been used as models. Proinflammatory factors such as ROS, specific cytokines and toxins of enteropathogenic bacteria can act by disrupting the tight junction and thereby compromising the intestinal barrier function. Tumor necrosis factor alpha (TNF-α) induces intestinal barrier impairment both *in vitro* and *in vivo* [53,54]. *In vitro*, TER could be used as a means of detecting intestinal barrier dysfunction of cell monolayers. Decrease in TER and increased permeability has been associated with down-regulation of ZO-1 and changes in the junctional localization [55], partly depending on increased expression and activation of the myosin light-chain (MLC) kinase protein [56]. *L. plantarum* ATCC 8014 was shown to inhibit the TNF-α-induced disturbance of TER as well as the increased IL-8 secretion in Caco-2 cells. Moreover, activation of extracellular signal-regulated kinase (ERK) and degradation of transcription factor NF-κB inhibitor IκB-α was counteracted by this strain [57]. Four different strains of lactobacilli (*L. delbrueckii* subsp. *bulgaricus* No. 3, *L. casei* No. 3, *Lactobacillus gasseri* No. 10, and *L. rhamnosus* OLL2838) were tested in a similar Caco-2 model and only the *L. rhamnosus* strain gave significant normalization of TER and decrease of IL-8 secretion as compared to TNF-α-induced cells without any treatment [41]. These authors also tested the supernatant of *L. rhamnosus* OL2838 but no effect on TER or IL-8 was obtained. On the other hand, when *L. rhamnosus* GG was challenged in Caco-2 cells treated by hydrogen peroxide where this compound destroys TER and increases permeability, it became clear that secreted proteins of this strain were effective against the insult. They prevented the hydrogen peroxide-induced redistribution of occludin, ZO-1, E-cadherin, and β-catenin from the intercellular junctions. This effect was brought about by the activation of ERK1/2 and protein kinase C isoforms PKCβ1 and PKCε [58].

Infection by pathogenic *E. coli* (EPEC) increases paracellular permeability of colonic cells. Philpott *et al.* [59] recorded a decrease in TER and a change in ZO-1 distribution in T84 cells three hours after infection, and later it was shown that EPEC-induced MLC-phosphorylation was involved in this disturbance [60]. *L. casei* DN-114001 inhibited, in a dose-dependent manner, such effects on TER and ZO-1 both when coincubated and when added three hours after infection [61]. A preventive effect on EPEC-induced increase in short-circuit current has also previously been described for *L. plantarum* 299v [62]. For enteroinvasive *E. coli* (EIEC), another strain of the latter species (*L. plantarum* CGMCC No. 1258) counteracted both the decrease of TER and increase in permeability of the dextran probe that was brought about by this infection on Caco-2 cells. The lower expression, as well as the redistribution of claudin-1, occludin, JAM-1, and ZO-1, was prevented. In fact, the *L. plantarum* strain itself made the tight junction proteins concentrate at the cellular contact sites where they are known to stabilize the integrity of Caco-2 cells. Moreover, *L. plantarum* CGMCC No. 1258 reversed the EIEC-induced alteration of peri-junctional actin filaments [63].

## 4. Effects in Human Studies

Although there is impressive evidence of lactobacilli influencing paracellular permeability in animal models and cell systems, studies of such effects in humans are rare. However, in a very recent publication, a small but elegant randomized, crossover study in humans showed that *L. plantarum* WCFS1 affected relocation of ZO-1 and occludin of duodenal cells [15], in accordance with that of *L. plantarum* CGMCC No. 1258 on Caco-2 colonic cells mentioned above [63]. More precisely, these proteins were found, by fluorescent staining of biopsies, to be significantly increased in the vicinity of the tight junction structures six hours after administration of the lactobacilli at this site as compared to administration of placebo in the same person. In Caco-2 cells, concomitant with activating TLR-2, *L. plantarum* WCFS1 was shown to protect against the drop in TER that was provoked by phorbol 12, 13 dibutyrate. This chemical is known to dislocate ZO-1 and occludin from the tight junction area. Activation of TLR2 by a TLR2 agonist attenuated the effects of the phorbol ester, just as did the *L. plantarum* strain. The authors conclude that their findings “provide a highly plausible explanation for the reported effects of *L. plantarum* and other probiotic strains on barrier disruption by inflammatory cytokines, chemicals and infection agents” [15]. 

This conclusion is very likely to be true for such a rapid effect on permeability as the one described above. However, other mechanisms could be at work, especially in a longer time frame. *L. plantarum* 299v has been shown to decrease IL-8 production of colonic epithelial cells while adhering to mannose at the surface of those [64]. This may counteract infiltration of neutrophils and thereby increased paracellular permeability. The adhesion property of this strain has also been seen to play a pivotal role in reducing bacterial translocation in endotoxemic rats [65]. Specific strains, also when adhering to epithelial cells, upregulate production of MUC3 mucin that, in turn, prevents pathogenic *E. coli* from attaching and from executing its effects on paracellular permeability [62,66,67]. Moreover, effects by lactobacilli on the very composition and/or activity of the entire microbiota might, in this complex ecosystem, affect the paracellular permeability.

### Clinical Trials

Clinical trials challenging the effect of probiotics on intestinal permeability are few and sometimes inconclusive. In a prospective randomized trial in critically ill patients, *L. plantarum* 299v was given by enteral feeding as compared to conventional therapy. No differences between the groups were found in terms of septic morbidity or mortality and only a trend towards lower intestinal permeability was recorded as measured by lactulose/rhamnose ratio for the group receiving *L. plantarum*. However, at fifteen days of treatment, the serum IL-6 was significantly lower in the probiotic group [68]. Furthermore, in a placebo controlled study of critically ill patients treated by antibiotics, *L. plantarum* 299v was shown to reduce the colonization of *Clostridium difficile* and, in a sub-group of patients in this study, the lactulose/rhamnose excretion ratio was monitored and found to be improved in the group receiving the probiotic as compared to the placebo group [69]. One reason for the conflicting results could be that in the latter study, the daily dose of the probiotic bacteria was approximately 10-times higher than in the previously described study.

The paracellular permeability of colonic biopsies from patients with irritable bowel syndrome (IBS) turned out to be increased compared to those from healthy persons. This was associated with a lower expression of ZO-1 mRNA in biopsies of IBS. When soluble factors from the biopsies were harvested, those from IBS patients decreased ZO-1 expression and increased TER in Caco-2 cells as compared to those from healthy subjects. This effect on increased paracellular permeability was positively correlated with severity of abdominal pain of the participants [19]. *L. plantarum* 299v has been administered in randomized, double blinded, placebo controlled trials to IBS patients, where they experienced relief in their symptoms including significantly less bloating and abdominal pain [70,71].

In children, the effect of *L. rhamnosus* GG has been addressed in two different patient groups, namely children with atopic dermatitis and children with short bowel syndrome. In a double-blinded, placebo-controlled, cross-over study, intake of this probiotic meant that the children with atopic dermatitis experienced fewer gastrointestinal symptoms. At the end of the study, the lactulose to rhamnose ratio was lower in the probiotic group than in the placebo group, and a positive correlation was found between this ratio and the severity of eczema [72]. In contrast, no significant effect on the lactolose to mannitol ratio was obtained in the trial done on children with short bowel syndrome [73].

## 5. Conclusions

Maintaining paracellular permeability in the gut at a normal level is a very central function that is compromised in many disease situations. Already 15 years ago, certain strains of lactobacilli were shown to have the ability to counteract the increased intestinal permeability provoked by methotrexate in the rat. Since then, a lot of data along these lines has been gathered, mainly in different animal models and *in vitro* cell systems. Although probiotics could contribute to the overall intestinal barrier function by increasing mucin production, by immunomodulation, by pathogen inhibition and by influencing the entire microbiota, the time has come to really challenge the effect of probiotics on paracellular permeability *per se* in humans. 
